# The anterolateral approach to the proximal humerus for nonunions and delayed unions

**DOI:** 10.4103/0973-6042.80466

**Published:** 2011

**Authors:** Carolyn M. Hettrich, Omesh Paul, Andrew S. Neviaser, Emily A. Borsting, Dean G. Lorich

**Affiliations:** Department of Orthopaedic Trauma Hospital for Special Surgery, New York, USA

**Keywords:** Nonunion, proximal third humerus fractures

## Abstract

Nonunions of proximal humerus fractures can be disabling as a result of pain, deformity and instability, and are often found in geriatric patients with poor bone quality. There are relatively few studies examining the treatment of nonunions of the proximal third of the humerus and the ideal treatment and surgical approach remains unclear. This case series reports the successful use of the anterolateral acromial approach for treatment of the symptomatic proximal third humerus nonunions in a geriatric group of patients with clear challenges as a result of patient comorbidities and bone quality.

## INTRODUCTION

Proximal humerus fractures account for approximately 5% of all fractures. However, the incidence is increasing with aging of the population.[1] Eighty percent of these fractures heal with nonoperative treatment measures; however, complex, unstable and severely displaced injuries can result in nonunion, leading to pain, deformity and pseudoarthrosis.[2] Decreased use can result in secondary adhesive capsulitis. Pain can limit the elderly patients’ ability to perform activities of daily living (ADL) and threaten their ability to function independently.[2–4] Outcome of treatment varies according to the fracture pattern, fracture location and the ability of the patient to recuperate from the injury and surgery.[5–9]

There are relatively few studies examining the treatment of nonunions of the proximal third of the humerus, and the ideal treatment remains unclear. Surgical fixation can be technically challenging due to osteoporotic bone, decreased shoulder range of motion and pseudoarthrosis.[2–4] Multiple techniques and different surgical approaches have been described. Treatment options include intramedullary roding, rods with tension bands, open reduction and internal fixation, prosthetic replacement and use of intramedullary cortical bone grafts.[3,6,8,10–14] Locking plate fixation has emerged as the preferred method for osteosynthesis of proximal third humerus fractures in osteoporotic bone.[15]

Surgical approach and fixation should address osteopenia as well as the characteristics of the fracture. Complex proximal humeral fractures with excessive comminution and distraction are frequently associated with severe soft tissue damage. Blood supply to the proximal segment of the humerus may be compromised, leading to delayed healing and development of osteonecrosis or atrophic nonunion.[5,7,16–18] Nayak *et al*. reported on 17 patients with severely displaced and unstable proximal humerus nonunions, with a 20% incidence of persistent nonunion and avascular necrosis (AVN) after treatment.[10] Kristiansen and Christensen reported a 13% incidence of AVN in patients treated for severely displaced proximal humerus fractures.[19]

This case series reports the successful use of the anterolateral acromial approach for treatment of the symptomatic proximal third humerus nonunions in a geriatric group of patients with clear challenges as a result of patient comorbidities and bone quality.

## CASE 1

**Figure 1:**
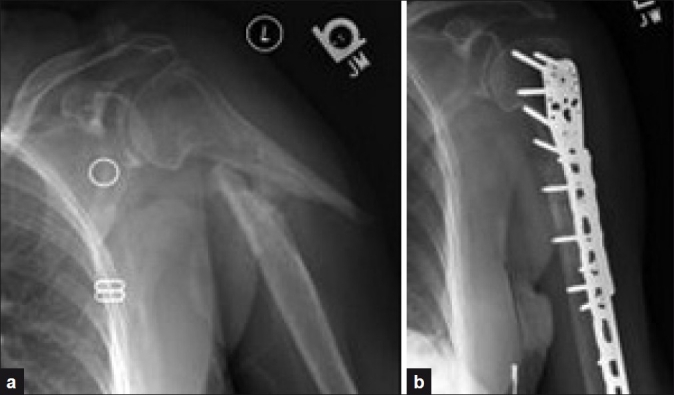
(a) Pre- and (b) post-operative radiographs of patient #1

A 72-year-old female presented 14 months after a two-part left proximal humerus diaphyseal fracture (OTA11 B2). Her past medical history was significant for osteoporosis. Physical examination revealed gross deformity, tenderness and motion at the fracture site. No neurologic deficit was found. Radiographic evaluation revealed an oblique fracture of the left proximal humerus diaphysis with lateral and anterior angulation of the distal fracture fragment [Figure 1a]. There was minimal osseus bridging between the proximal and distal fracture fragments with associated callus formation. She was indicated for operative intervention.

The patient was administered regional anesthesia and positioned in a sloppy lateral position. An extended anterolateral approach was utilized as described by Gardner *et al*.[20–22] The skin incision began at the anterolateral tip of the acromion and was extended to approximately 5 cm proximal to the lateral epicondyle. Proximally, the subcutaneous tissue was divided to expose the raphe between the anterior and the middle heads of the deltoid. The raphe was carefully divided in the “safe zone” above the location of the axillary nerve, 6 cm below the anterolateral border of the acromion.[20–21] The muscle was separated using blunt dissection proximally. The axillary nerve was then palpated and found to be scarred to the humeral head. Neurolysis was performed and the nerve and surrounding soft tissues were protected with a vessel loop. The deltoid was further divided proximal and distal to the nerve and the insertion site was tunneled under for future plate placement. The lateral border of the biceps was then identified and the muscle retracted medially to expose the brachioradialis and brachialis muscles. The faschia between the muscles was then incised in line with the intermuscular plane and the radial nerve was identified. The radial nerve was found to be scarred down to the inferior aspect of the fracture, and was mobilized. The fracture site was then exposed and debrided. A proximal humerus Locking Compression Plate (LCP) (Synthes, Paoli, PA, USA) was passed under the axillary nerve and down the lateral aspect of the humerus. The humerus was transfixed distally using two compression screws. An indirect reduction was obtained proximally. The obliquity of the fracture was clamped and compressed using a standard reduction clamp and locking screws were then placed proximally. An interfragmentary screw was then passed across the obliquity of the fracture from the lateral to the medial side. Anteriorly, a six-hole recon plate was placed with two screws proximally and two screws distally to reinforce the fixation. The glenohumeral joint was then examined and found to have significant bursal scarring, which was excised producing a full range of motion at the shoulder. Recombinant human bone morphogenetic protein-2 (Infuse Bone graft, Medtronic Inc., Minneapolis, MN, USA) was then packed around the fracture site. Aggressive occupational therapy for active and passive range of motion was implemented 1 week after surgery. At the patient’s 3-month follow-up, healing was confirmed radiographically and the fracture site was nontender [Figure 1b]. Range of motion for forward flexion, abduction and internal and external rotation at her 12 ^th^-month visit was 90, 90, L2 and 40 degrees, respectively [Table 1].

**Table 1:** Range of motion at last follow-up appointment for the uninvolved (U) and involved (I) shoulder

	Case I		Case II		Case III	
	U	I	U	I	U	I
Flexion	140	90	130	100	160	150
Abduction	135	90	110	90	140	140
Internal rotation	NR	NR	L1	L4	T8	T9
External rotation	50	40	50	50	70	60

## CASE 2

A 94-year-old female fell sustaining a right femoral neck and a four-part proximal humerus diaphyseal fracture (OTA 11B2). The patient underwent hip hemi-arthroplasty, but due to the advanced age and medical co-morbidities, the fracture of her humerus was treated in a Sarmiento fracture brace. Three months later, she continued to have persistent pain at the fracture site.

On physical examination, the arm was grossly deformed and there was tenderness and gross motion of the fracture. She had no neurological deficit. Radiographic evaluation showed completely displaced right oblique four-part proximal humerus diaphyseal fracture with minimal callus formation. Because of pain and inability to perform ADL, the patient requested surgical management.

The extended anterolateral approach was again utilized to expose the axillary and radial nerves as well as the fracture site in the same manner as described above. The radial nerve was found to be adherent to the posterior spike of the humerus requiring release. There was significant osteopenia and bone loss at the site of the fracture. A 10-cm fibular allograft was inserted as an intramedullary strut into the humerus. After the fracture was reduced using fluoroscopy, the proximal and distal fracture fragments were compressed over the allograft. A proximal humerus eight-hole locking plate (Synthes) was inserted and the distal fracture fragment was fixed to the plate. When the proximal segment was reduced to the plate, a unicortical fracture occurred anteriorly. This was provisionally held with a reduction clamp. Compression screws were then placed across the fracture site as well as locking screws proximally, two of which passed through the fibular strut. Distally, screws were also passed through the allograft. A 10-hole 2.4 LCP plate was then placed over the anterior crack and transfixed. The wound was then irrigated and closed in the same manner as above. A plaster posterior splint was fabricated to maintain the arm in flexion and then was placed into a sling. Aggressive active and passive range of motion was instituted on POD #1. The patient recovered from the operation without incident. Complete union with significant callus formation was confirmed on radiographic evaluation at the 3-month follow-up. At 1-year follow-up, her range of motion for forward flexion, abduction and internal and external rotation was 100, 90, L4 and 50 degrees, respectively [Table 1].

## CASE 3

**Figure 2:**
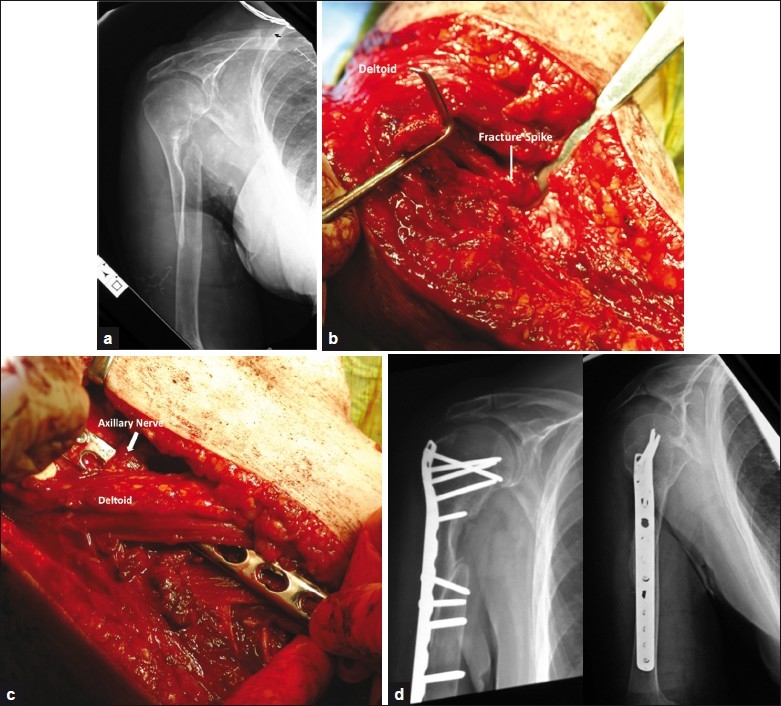
(a) Preoperative radiographs of the patient in case 3; (b) Intraoperative image depicting the lateral surgical approach, with the fracture site identified, prior to debridement; (c) Intraoperative photograph of tunneling the plate under the axillary nerve and the deltoid insertion; (d) Postoperative radiographs

A 75-year-old female patient presented to the clinic after 6 months of an oblique, two-part, right proximal humeral diaphyseal fracture (OTA 11 B2), complaining of pain and disability. She had initially been managed nonoperatively due to medical co-morbidities, which included a 30-year history of insulin-dependent diabetes mellitus and significant osteoporosis. On physical examination, there was a visible deformity of the arm. Motion was severely limited due to pain. There was also weakness of wrist extension, suggesting a radial nerve deficit. Radiographs revealed an oblique proximal humerus nonunion [Figure 2a].

The extended anterolateral acromial approach was used. The radial nerve was found to be scarred to the fracture site, and was released. The fracture site was exposed and the fibrous psuedocapsule was opened [Figure 2b]. The fragments were found to be entwined in the biceps. The biceps margin was partially released to allow mobilization. The pectoralis major tendon was attached to the proximal fragment and 1 cm of the distal insertion was released to again allow for mobilization. A locking LCP metaphyseal plate (Synthes) that was contoured to match the curve of the greater tuberosity was passed deep to the axillary nerve and fixed proximally with five locking screws [Figure 2C]. The distal fragment was held to the plate with a Verbugge clamp and four locking screws were then placed into the distal fragment. Postoperative radiographs can be seen in Figure 2d. Two 4.5 mm cortical screws were placed across the fracture site for compression. Fifty cc of DBX Putty (Synthes) was placed along the nonunion medially and laterally. Postoperatively, the operated arm was kept in a sling. Active and passive range of motion was started after 3 days. Fracture union with significant callus formation was confirmed radiographically on the 3-months follow-up visit. At her 1-year follow-up visit, the range of motion for forward flexion, abduction and external and internal rotation was 150, 140, T9 and 60 degrees, respectively [Table 1].

## DISCUSSION

Nonunion of proximal humerus fractures can be disabling as a result of pain, deformity and instability. Symptomatic cases require surgical intervention. Factors such as osteopenia/osteoporosis, pseudoarthroses and joint adhesions can create a challenge even for an experienced surgeon. Outcomes for surgical management for proximal humerus nonunion have been reported, with failure rates ranging from 9% to 20%[4,8,10] An optimal surgical approach would provide adequate exposure of the fracture fragments and relevant anatomy while preserving the tissue viability with minimal dissection to achieve higher rates of union.

The standard deltopectoral approach is widely used for fixation of the proximal humeral fractures, and has been associated with damage to the critical branches of the anterior and posterior circumflex vessels.[16,21,23] In addition, this anterior approach requires significant soft tissue dissection and muscle retraction to ensure adequate exposure for reduction of the fracture. Lateral plating may further compromise the blood supply and tissue viability. Recognition of the importance of soft tissue preservation has led to minimally invasive surgical techniques.[22,23] This is especially important in treating nonunions as healing depends on restoration of local biology.[2,15]

The standard deltopectoral approach is an indirect (anterior) approach to the lateral plating zone. This approach can result in prolonged muscle retraction to access the posterolateral fracture fragments of the humeral head in complex three- and four-part fractures. In addition, access to the greater tuberosity requires elevation of a portion of the insertion of the deltoid. Klepps *et al*. in their cadaveric study on deltoid muscle described that release of even one-fifth of its insertion can lead to significant weakness.[24] Lateral extension of this approach puts the circumflex vessels and its branches at risk.[21–25] In a study comparing the minimally invasive technique with the open technique, Bathis *et al*. reported a 16% incidence of AVN with the open technique as compared with 9% with the minimally invasive technique using the minimal anterolateral approach.[26] Struzenegger *et al*. reported minimal tissue disruption while fixing nonunions is associated with higher rates of union.[7]

In a previous study, we described a minimally invasive anterolateral approach for complex proximal humerus fractures.[22] This approach was previously restricted due to the position of the axillary nerve for its use for only up to 3-5 cm distal to the acromion.[9,20–23] With an accurate knowledge of anatomy, the axillary nerve can be palpated and easily protected.[27] Meyer described an avascular bare area, an approximately 3-cm-wide region between the penetrating humeral head vessels.[17–27] The extended anterolateral approach has an advantage of direct access to the fracture site, permitting mobilization and reduction of the fracture fragments with minimal soft tissue stripping of the proximal and distal fracture fragments. Further, this is a direct approach to the region, laterally, for optimal placement of the anatomically designed hardware used in the treatment of proximal humerus fracture and nonunion. An additional benefit to this approach is an easy extension of exposure to more distal fracture fragments as well as the radial nerve.[25] In our previous study of 52 patients treated using the extended anterolateral approach, we reported no cases of AVN or neuropraxia postoperatively. A recent comparison of outcomes between deltoid splitting (DS) and deltopectoral (DP) approaches revealed a lower rate of AVN in the DS group.[28] In addition, patients in the DS group had more shoulder strength and fewer complaints of pain and impingement.

In our case series, we used the extended anterolateral approach to expose the non- or delayed union sites as well as the axillary and radial nerves. Locked plate fixation through this approach avoids unnecessary handling of the soft tissues and gives direct access to the lateral zone of the proximal humerus. This approach does not require release of deltoid insertion, reducing postoperative deltoid weakness. We achieved union in all patients by 3 months. Additionally, in this series, we were able to successfully treat a case of associated neuropraxia. At 1-year follow-up, the patients’ range of motion was comparable to the contralateral side, and all were able to perform their ADL. We believe that the anterolateral approach is superior to the standard deltopectoral approach for the treatment of proximal humeral nonunion because it requires less soft tissue dissection, obviates the need to release the deltoid insertion and allows more direct access to the lateral surface of the humerus.

### Surgical pearls

- Leave a cuff of soft tissue surrounding the axillary nerve for additional protection
- Do not use screw guide with the plate as this puts too much stretch on the axillary nerve
- Identify and mobilize the radial nerve prior to exposure and debridement of the fracture site
- Rigid fixation allows for early postoperative range of motion to prevent arthrofibrosis
